# Light Emission Properties of Thermally Evaporated CH_3_NH_3_PbBr_3_ Perovskite from Nano- to Macro-Scale: Role of Free and Localized Excitons

**DOI:** 10.3390/nano12020211

**Published:** 2022-01-10

**Authors:** Claudia Triolo, Maria Luisa De Giorgi, Antonella Lorusso, Arianna Cretì, Saveria Santangelo, Mauro Lomascolo, Marco Anni, Marco Mazzeo, Salvatore Patané

**Affiliations:** 1 Department of Civil, Energy, Environmental and Materials Engineering (DICEAM), Mediterranean University, 89122 Reggio Calabria, Italy; saveria.santangelo@unirc.it; 2 Dipartimento di Matematica e Fisica “Ennio De Giorgi”, Università del Salento, 73100 Lecce, Italy; marialuisa.degiorgi@unisalento.it (M.L.D.G.); antonella.lorusso@unisalento.it (A.L.); marco.mazzeo@unisalento.it (M.M.); 3 IMM-CNR Institute for Microelectronic and Microsystems, Via per Monteroni, 73100 Lecce, Italy; arianna.creti@cnr.it (A.C.); mauro.lomascolo@cnr.it (M.L.); 4 CNR NANOTEC—Institute of Nanotechnology, 73100 Lecce, Italy; 5 Department of Mathematical and Computer Sciences, Physical Sciences and Earth Sciences, University of Messina, 98166 Messina, Italy; salvatore.patane@unime.it

**Keywords:** perovskite, thermal evaporation, trap states, free excitons, localized excitons, PL emission, SNOM, ASE

## Abstract

Over the past decade, interest about metal halide perovskites has rapidly increased, as they can find wide application in optoelectronic devices. Nevertheless, although thermal evaporation is crucial for the development and engineering of such devices based on multilayer structures, the optical properties of thermally deposited perovskite layers (spontaneous and amplified spontaneous emission) have been poorly investigated. This paper is a study from a nano- to micro- and macro-scale about the role of light-emitting species (namely free carriers and excitons) and trap states in the spontaneous emission of thermally evaporated thin layers of CH_3_NH_3_PbBr_3_ perovskite after wet air UV light trap passivation. The map of light emission from grains, carried out by SNOM at the nanoscale and by micro-PL techniques, clearly indicates that free and localized excitons (EXs) are the dominant light-emitting species, the localized excitons being the dominant ones in the presence of crystallites. These species also have a key role in the amplified spontaneous emission (ASE) process: for higher excitation densities, the relative contribution of localized EXs basically remains constant, while a clear competition between ASE and free EXs spontaneous emission is present, which suggests that ASE is due to stimulated emission from the free EXs.

## 1. Introduction

Metal halide perovskite semiconductors have attracted extensive attention for their outstanding optoelectronic properties [1,2,3,4], such as high defect tolerance, long carrier lifetime and diffusion length, and tunable optical bandgap across the whole visible spectrum. In addition, halide perovskites can be easily processed using cost-effective and low-temperature solution fabrication, i.e., thermal evaporation [5,6] and vapor phase [7,8] deposition. Due to these features, perovskites found large application in photovoltaic cells [6,9], light-emitting diodes [10,11,12], and optically pumped lasers [13,14,15]. For these devices, the study and individuation of the emission mechanisms are crucial issues to determine the channels for the photogenerated species loss, the spectral properties of the device emission, and the factors affecting the stimulated emission threshold.

Based on the most recent literature reports [16,17,18], the band-to-band transition (also known as free-carrier recombination, FC), excitons (EXs), and trap states (TSs) contribute to the spontaneous emission in the perovskites. Among them, the recombination of FC and EXs are competitive mechanisms in the spontaneous emission process, and their relative amount affects the spectral features of the optical emission. The correlation between emission mechanisms and processing conditions, as well as the material composition, environment [19], and photoinduced degradation [20], still represent open questions for these materials.

In order to cope with these inherent features in perovskites and improve their emission quantum yield, many strategies have been developed [21,22,23,24,25], including chemical and physical approaches to passivate non-radiative defects (usually associated with the presence of deep TSs in the bandgap), thus favoring the radiative recombination channels. We recently demonstrated [26] that the equilibrium condition between photoexcited species densities, quantitatively expressed by Saha’s equation [27], can be modified by the TSs passivation obtained by irradiating the sample by means of a UV laser under wet air conditions: this results in increasing electron–hole pairing to form bound neutral states, EXs thus becoming the dominant species. Nevertheless, the differences in the role of free and localized excitons are still unclear, and thermally deposited perovskites have still not been studied.

In the present paper, the optical emission properties of thermally evaporated thin films of CH_3_NH_3_PbBr_3_ perovskite are analyzed exploiting the scanning near-field optical microscopy (SNOM) technique, allowing us to correlate the spontaneous emission to the morphological properties of the perovskite layer at a nanoscale. Due to its very high spatial resolution [28,29,30,31], PL-SNOM (photoluminescence-SNOM) imaging allows optically resolving nanoscale structures, at the same time collecting the optical and topography maps for a direct correlation between the near-field PL signal and the sample morphology. A detailed line-shape analysis of the local PL emission allows us to locally quantify the contributions of the FC, EXs, and TSs to the spontaneous emission and to investigate their spatial distribution on the single crystallite grains of the film. Specifically, we demonstrate that in the case of thermally deposited perovskites, EXs are the main species contributing to the spontaneous emission. The nanoscale analysis of spontaneous emission also clarifies the spatial and spectral distribution of free and localized EXs on the emission properties. Moreover, we analyzed the contribution of the free and localized excitons as well as the role of the TS on the amplified spontaneous emission (ASE), which is crucial for the development of semiconductor lasers based on perovskites. Our experiment demonstrates that the control of the film morphology is crucial to determine the processes affecting the emission and, thus, to control the emission properties of the active films.

## 2. Materials and Methods

### 2.1. Sample Preparation

We deposited CH_3_NH_3_PbBr_3_ films on glass substrates. The perovskite films were fabricated by co-depositing, in a vacuum chamber (10^−7^ mbar), the precursors methylammonium bromide and lead (II) bromide from two independent sources. The stoichiometry of the two perovskite precursors was MABr:PbBr_2_ = 1:1, co-evaporated at a rate of 0.3 Å s^−1^ (reached at about 150 °C) and 0.25 Å s^−1^ (reached at about 300 °C), respectively. Perovskite film was not annealed after the deposition.

### 2.2. Characterization

The atomic force microscopy (AFM) measurements are performed in semi-contact mode using a silicon cantilever mounted on a NT-MDT microscope (Smena Head). SNOM measurements are carried out in transmission mode with hollow-pyramid cantilever working in contact mode. An unpolarized solid-state laser (λ_exc_ = 470 nm) coupled to a NT-MDT Integra Spectra C microscope is used as an excitation source. A 100X objective (Mitutoyo, NA = 0.70) focuses the laser beam on the nanohole of the cantilever. The transmitted light from the sample, collected by an optical fiber (THORLABS), is detected by a PMT (HAMAMATSU) coupled with it.

Micro-PL measurements are performed in reflection mode using the same setup (NT-MDT Integra Spectra C). The incident laser (λ_exc_ = 470 nm) and the PL signals from the sample surface pass through the 100X objective. Finally, the PL signal is dispersed by an optical grating (600 lines/mm), placed inside a monochromator (SOLIS), and it is detected by a cooled CCD Camera (Andor IDus). PL measurements during CW laser irradiation in air are performed by exciting the sample with a UV laser diode at 405 nm, with a power up to 100 mW, with a spot of about 2 mm.

The amplified stimulated emission (ASE) measurements were performed by exciting the film by a LTB MNL 100 nitrogen laser, delivering 3 ns pulses at 337 nm, with a peak energy up to 155 µJ, focused by a cylindrical lens on a rectangular stripe with length of 1.7 mm and width of 80 µm. The laser excitation density is varied by a variable neutral filter. In both cases, the sample emission is collected from the sample edge, after waveguiding along the film, spectrally dispersed by an Acton 750 spectrometer, and detected by a Andor Peltier cooled CCD. The spectral resolution is about 1 nm.

## 3. Results and Discussion

**Figure 1:**
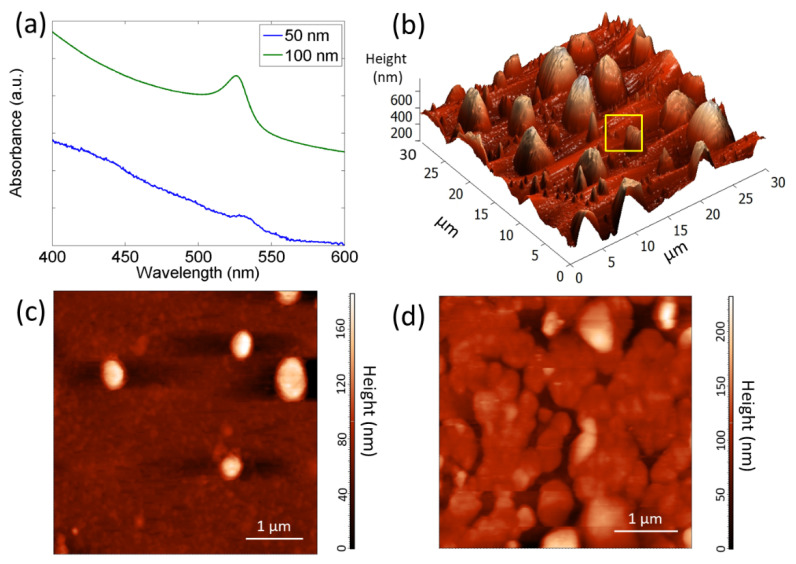
(**a**) Absorbance spectra (in a.u.) of the perovskite thin films with nominal thickness of 50 nm (sample A) and 100 nm (sample B). In order to distinguish the two spectra, that of sample B is up-shifted along the *y*-axis; (**b**,**c**) AFM images of sample A and (**d**) sample B. Panel c shows a detail of the bottom layer of sample A, marked by the yellow square in panel b.

Thin films of CH_3_NH_3_PbBr_3_, with two different nominal thicknesses (sample A, 50 nm, and sample B, 100 nm), are obtained via thermal evaporation on glass substrate. The absorption spectra of the two samples as well as their surface morphologies are shown in Figure 1. The spectra of the samples are characterized by an excitonic peak which blue-shifts from 532.0 ± 0.5 nm to 526.0 ± 0.5 nm with increasing thickness of the deposited material [26,32]. Figure 1b–d display the surface morphology of the two samples. We observe that the nominal thickness (amount of deposited material) of the films heavily influences their surface morphology.

In particular, the thinner sample (Figure 1b,c and SEM image in Figure S1a) consists of crystallites with a pyramidal-like structure, grown over a compact and smooth bottom layer with an average roughness of about 1.8 nm. The lateral sizes of crystallites vary in the range of 0.2–4 μm while the height of larger crystallites reaches about 700 nm. The pyramidal shape is due to the self-assembly process, already reported for the perovskite nanoplatelets [33]. The apparent discrepancy between the nominal thickness of the deposited film, as estimated by the sensor placed inside the vacuum chamber and by AFM, is attributed to the self-assembly of the crystallites, which results in a jagged surface morphology with crystallites whose height frequently exceeds 100 nm.

Sample B is characterized by an almost homogeneous surface, constituted of micro-crystals, about 1.5 μm in diameter, which form greater islands coalescing among each other (Figure 1d and SEM image in Figure S1b). The average surface roughness, calculated from the AFM image analysis, is about 28.9 nm.

The different morphologies of the two investigated samples, in terms of homogeneity of the crystallite size, are responsible of the variations observed in the absorption spectra, where the EX peak is characterized by a different wavelength, intensity, and shape.

The emission properties of the samples are investigated after the exposure to UV radiation (405 nm), CW laser intensity of 30 mW mm^−2^ in wet air. This procedure promotes the removal of the shallow TSs, favoring the photogenerated carries to radiatively recombine [21]. In order to identify the emissive species and to correlate the emission properties to the surface morphology at a nano- and micro-scale, we performed SNOM measurements.

**Figure 2:**
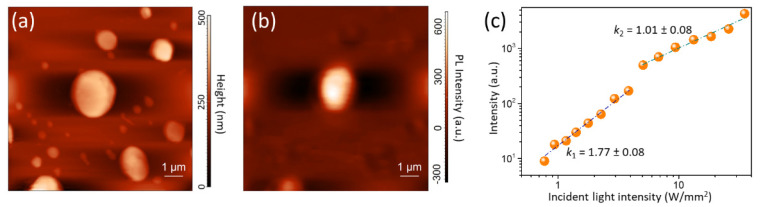
(**a**) Morphology and (**b**) PL-SNOM image of sample A acquired in transmission mode; (**c**) PL intensity in sample A as a function of the fluence of the incident radiation, acquired in transmission mode. The dashed lines are the results of the linear regression procedure using a power-law function for each selected range of the incident light intensity.

The comparison between morphological and PL-SNOM images (Figure 2a,b) evidences that the whole sample surface emits a PL signal in the visible range, with higher intensity in correspondence with the center of the larger crystallite. In order to investigate the nature of the dominant emission species, we measured the PL intensity dependence on the incident radiation power density (Figure 2c). The results evidenced the existence of a power-law dependence [34] with an exponent k_1_ = 1.77 ± 0.08 below 5 W mm^−2^ and k_2_ = 1.01 ± 0.08 at higher incident energy power density. This behavior can be understood in terms of a multichannel dynamics and kinetics of light emitting species. At low incident intensity, the photo-excited FC can be partially trapped by un-passivated TSs [34,35]; on the contrary, at higher intensity, TSs are almost completely passivated and an improved FC-to-EXs gemination rate occurs [26], thus resulting in a linear power-law dependence.

**Figure 3:**
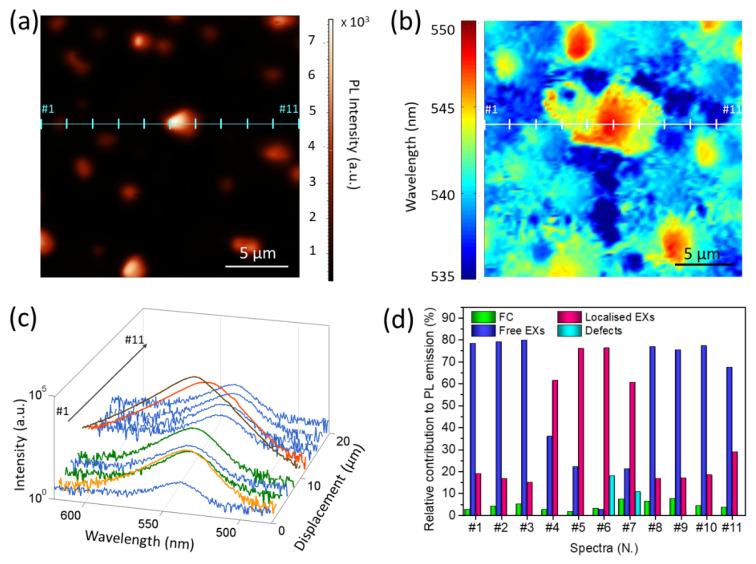
(**a**) Micro-PL intensity map of sample A acquired in reflection mode; (**b**) map of the wavelengths of the maximum PL emission, as derived from micro-PL map in sample A (panel a); (**c**) PL spectra, recorded in sample A along a selected direction (signed by the cyan line in panel a) acquired at different locations, with 2 μm spacing distance from each other; the same color as in panel b are used. For a better comparison, a logarithmic scale is selected to show their intensity; (**d**) relative amount of FC, free, localized EXs, and defects estimated as fractional area contribution of each emissive species for each spectrum shown in panel c. All data, including the real counts of the acquired optical signal in correspondence with the wavelength emission, are reported in Table S1.

In order to gain a deeper insight into the local emission properties of the samples and in the relative emitting species contribution, we also performed micro-PL measurements (λ_exc_ = 470 nm, 1 W mm^−2^), thus determining both the integrated local emission intensity and the local PL spectra. The obtained intensity map of sample A (Figure 3a) confirms that the whole sample surface emits light in the visible range and the PL signal intensifies in correspondence of the larger grains, as already observed for the SNOM images. In addition, these measurements allow us to observe that the distribution of the PL emission peak wavelength (Figure 3b), obtained from a post-acquisition analysis of Figure 3a, is not spatially uniform over the sample surface.

In particular, we observe that the entire bottom layer shows a homogeneous emission at wavelengths in the range 535–540 nm, while a clear red-shift is observed in correspondence with the grains, up to 550 nm in the center of the larger grains. A further insight into the local PL emission properties is achieved by analyzing the micro-PL spectra (Figure 3c) collected in different position along the x-direction (cyan line in Figure 3a), revealing [26] that different species contribute to the spontaneous emission, namely the FC (~520 nm, or ~2.37 eV) [36], free (~536 nm, or ~2.30 eV), and localized EXs (~548 nm, or ~2.25 eV) [34]. Thus, as shown in Figure S2a, three Gaussian bands are used to reproduce the spectra. At some locations (#6 and #7), an additional Gaussian band is needed to reproduce the spectral profile at the longer wavelength (~ 565 nm) (Figure S2b). This additional contribution is ascribed to the recombination in TSs below the energy gap [37]. The presence of the optically active defects can be induced by the self-assembly process that generates the crystals, and it is typically associated [38] with emission from shallow trap levels in the band gap due to local structural deformations of the crystalline lattice [17] and/or punctual defects [39]. The plot of the relative intensity (fractional area) of the considered emissive species (Figure 3d) in the CWPL spectra indicates that the band-to-band transition poorly contributes to the PL emission, while the EXs are the dominant emissive species. In particular, the free EXs are responsible for the emission from the bottom layer (at about 539 nm), whereas localized EXs mainly contribute to very intense emission from crystallites (at about 545 nm).

A comparison between the normalized absorption and PL spectra (Figure S3) evidences a red-shift of the PL band with respect to the excitonic peak. This energy shift is attributed to the self-absorption due to the finite thickness of the crystallites [16] and to the presence of localized EXs, which mainly contribute to the PL emission, producing a remarkable energy shift.

As suggested by He et al. [34], the localized EXs are formed from the transfer of photogenerated carriers to the local potential minima states in the conduction and/or valence bands, known as tail states. The presence of the tail states can be induced by the compositional changes and structural deformations [17], which are more likely to be present inside the larger crystallites produced via the self-assembly process. Conversely, the compact bottom layer emits through recombination processes of the free EXs. Here, the emission process can be reasonably justified considering that the bottom layer contains a negligible (or absent) percentage of tail states, since the crystallite formation did not occur.

Similar results have been obtained for sample B. Figure S4 shows the PL intensity map and the PL spectra acquired on- and out-of-grain. The coalescence of grains and the increased thickness of the deposited film reflect into a greater number of bright spots in the map. As in the case of the thinner sample, the PL emission intensifies and shifts towards longer wavelengths in correspondence with the bigger grains (bright spots in Figure S4a).

In order to better evidence the mainly excitonic nature of the emitting species, the role of free and localized EXs in the emission properties and the effects of the photoinduced reduction of the trap density, the macroscopic photoluminescence and the ASE properties of the samples are also investigated. As ASE measurements require the collection of the sample PL emission from the film edge after waveguiding in the film, sample B is analysed due to its higher thickness and more uniform morphology.

Figure S5 displays the PL spectra of the sample B. They are characterized by a peak at 532 nm and a shoulder at about 550 nm, which evidences an emission dominated by free and localized EXs radiative recombination. In order to progressively decrease the trap density, this sample is also exposed to CW laser light in air [26]. The so-obtained progressive saturation of the traps reflects into a corresponding increase of the PL intensity (see inset in Figure S5).

**Figure 4:**
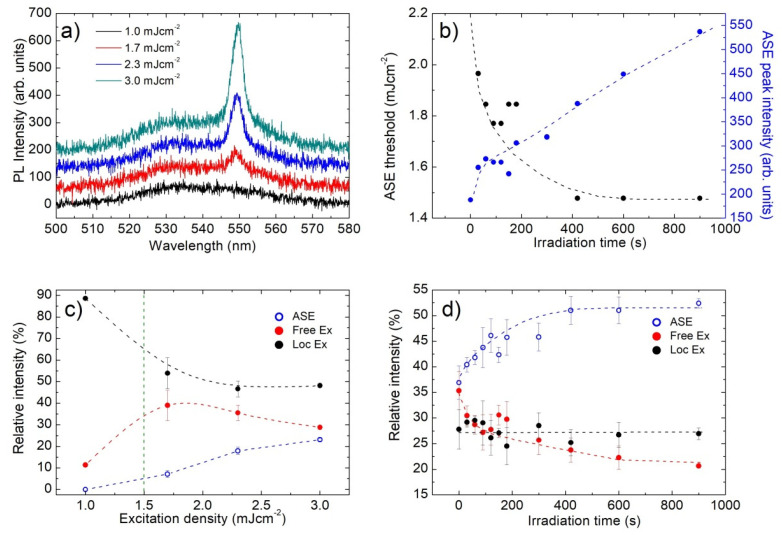
(**a**) PL spectra of sample B at the end of the irradiation process, as a function of the excitation density. The progressive appearance of ASE band about a wavelength of 550 nm is clearly visible; (**b**) ASE threshold (black dots) and ASE intensity at 3.0 mJ cm^−2^ recorded every 30 s of CW irradiation in air, showing a progressive threshold decrease and ASE intensity increase; (**c**) excitation density dependence of the relative contribution to the emission spectra of the ASE, free EXs and localized EXs bands (the lines are guides for the eyes); (**d**) irradiation time dependence of the relative contribution to the emission spectra of the ASE, free EXs and localized EXs bands (the lines are guides for the eyes).

When the sample is excited by a pulsed nanosecond laser in waveguide configuration, after the CW irradiation in air, a similar emission spectrum at low excitation density is observed (see Figure 4a). With increasing excitation density, a new narrow emission band at about 549 nm appears, which progressively dominates the emission. These features are qualitatively similar to previous results on CH_3_NH_3_PbBr_3_ films deposited from solution [40], thus allowing us to ascribe the additional emission band to ASE, with a threshold of about 1.5 mJ cm^−2^.

In order to directly probe the effects of the trap density on the ASE properties, these are investigated during the CW irradiation, by pumping the film in air, after CW irradiation steps of 30 s. For each irradiation time, the ASE threshold and the emission spectrum at a fixed excitation density of 3.0 mJ cm^−2^ are measured. The obtained results allow observing (see Figure 4b) a progressive increase of the ASE peak intensity and a gradual diminishing of the ASE threshold, from about 2.0 mJ cm^−2^ of the non-irradiated sample down to about 1.5 mJ cm^−2^ as the spontaneous emission intensity increase reaches the saturation regime.

Further insight into the species determining the ASE emission is obtained by determining, by a multi-Gaussian fitting of the spectra as well, the relative contribution of ASE and of the free and localized EXs spontaneous emission to the total emission intensity, as a function of both the excitation density and the irradiation time. The obtained results (see Figure 4c) allow to observe that, below the ASE threshold (green dotted vertical line in Figure 4c), the PL is mainly due to localized EXs emission. As the excitation density reaches the ASE threshold, the relative intensity of localized EXs decreases, while the one of free EXs increases. For higher excitation densities, the relative contribution of localized EXs basically remains constant, while a clear competition between ASE and free EXs spontaneous emission is present. This last feature evidences that the progressive increase in the ASE intensity, related to a decrease to the lifetime of the excited state involved in the stimulated emission process, also determines a decrease in the free EXs spontaneous emission to the whole PL intensity, and it is thus a clear indication that ASE is due to stimulated emission from the free EXs.

This conclusion is confirmed by the irradiation time dependence of the relative intensities (see Figure 4d), again evidencing a basically constant contribution of localized EXs and that the progressive increase in the ASE relative intensity takes place together with a corresponding decrease in the free EXs spontaneous emission.

Overall, these results clearly evidence the importance of reducing the trap density in order to improve not only the spontaneous emission but also the stimulated emission properties of the active thermally deposited film, as well as the importance of a good control of the film morphology in order to minimize the localized EXs contribution to the sample photoluminescence.

## 4. Conclusions

In summary, the role of free and localized EXs on the spontaneous optical emission properties in CH_3_NH_3_PbBr_3_ perovskite has been investigated at the micro-scale. The emission spectral features correlate with the morphology: the free EXs are the main emitters in the bottom layer, while the localized EXs are dominant in the crystallites, which cause a strong enhancement of the PL emission, while FCs play a minor role.

A confirmation of EXs as the main emitting species and the beneficial role of the TS-passivation procedure also emerges from the ASE analysis. The wavelength of the ASE band (549 nm) coincides with that observed for the excitonic emission in the micro-PL map. The relative contribution of localized EXs remains constant at higher excitation densities, while the competition between ASE and free EXs spontaneous emission indicates the free EXs as the main emitting species in the stimulated emission process from evaporated perovskites.

## Supplementary Materials

The following are available online at https://www.mdpi.com/article/10.3390/nano12020211/s1, Figure S1: SEM image of sample A and Sample B. Figure S2: Example of line-shape analysis of PL spectra. Figure S3: Normalized absorption and PL (out- and on- particle) spectra of sample A. Figure S4: Micro-PL intensity of sample B. Figure S5: PL spectra of sample B during continuous UV irradiation in air. Table S1: Wavelength and intensity of the maximum PL emission in the CWPL spectra.
