# Supplementary material for: Light Emission Properties of Thermally Evaporated CH3NH3PbBr3 Perovskite from Nano- to Macro-Scale: Role of Free and Localized Excitons
Source: Nanomaterials (Basel). 2022 Jan 10;12(2):211. doi: 10.3390/nano12020211 (PMC8779009; doi:10.3390/nano12020211)
Supplement: Supplementary file 1 [file nanomaterials-12-00211-s001.zip › nanomaterials-1544790-supplementary.pdf]

nanomaterials

Light emission properties of thermally evaporated $CH_3NH_3PbBr_3$ perovskite from nano- to macro-scale: Role of free and localized excitons

Claudia Triolo [1,*], Maria Luisa De Giorgi [2], Antonella Lorusso [2], Arianna Cretì [3], Saveria Santangelo [1], Mauro Lomascolo [3], Marco Anni [2,*], Marco Mazzeo [2,4], Salvatore Patané [5]

1 Department of Civil, Energy, Environmental and Materials Engineering, Mediterranea University, 89122, Reggio Calabria, Italy; saveria.santangelo@unirc.it
2 Dipartimento di Matematica e Fisica "Ennio De Giorgi", Università del Salento, 73100, Lecce, Italy; marialuisa.degiorgi@unisalento.it (M.L.DG.); antonella.lorusso@unisalento.it (A.L.); marco.mazzeo@unisalento.it (M.M.)
3 IMM-CNR Institute for Microelectronic and Microsystems, Via per Monteroni, 73100 Lecce, Italy; mauro.lomascolo@cnr.it (M.L.); arianna.creti@cnr.it (A.C.);
4 CNR NANOTEC—Institute of Nanotechnology, 73100, Lecce, Italy.
5 Department of Mathematical and Computer Sciences, Physical Sciences and Earth Sciences, University of Messina, 98166 Messina, Italy; salvatore.patane@unime.it

* Correspondence: claudia.triolo@unirc.it (C.T.); marco.anni@unisalento.it (M.A.)

Supplementary Information

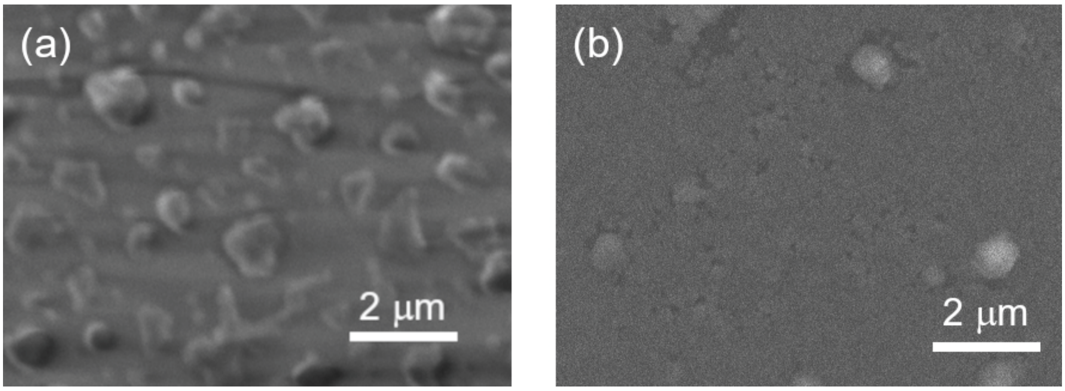

Figure S1. SEM image of sample A (a) and sample B (b).

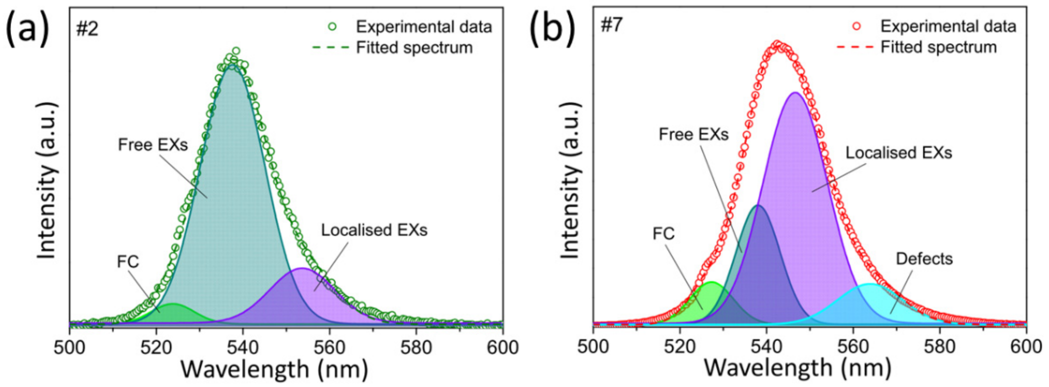

Figure S2. Line-shape analysis of PL spectra acquired at locations (a) #2 and (b) #7.

Table S1. Wavelength and intensity of the maximum PL emission in the CWPL spectra of Figure 3b. The relative amount of FC, free, localized EXs and defects, estimated as fractional area contribution of each emissive species are also reported

No.	Max of λ_{PL} (nm)	Max of I_{PL} (counts)	FC (%)	Free EXs (%)	Loc. EXs (%)	Defects (%)
#1	538.4	34	2.67	78.43	18.90	--
#2	538.4	435	4.13	79.10	16.77	--
#3	538.9	139	5.18	79.81	15.00	--
#4	543.4	88	2.52	35.97	61.51	--
#5	544.4	210	1.76	22.15	76.08	--
#6	547.4	22560	3.22	2.58	76.35	17.85
#7	542.9	4840	7.43	21.25	60.67	10.66
#8	537.9	53	6.37	76.95	16.67	--
#9	539.9	61	7.61	75.47	16.91	--
#10	537.9	81	4.35	77.29	18.35	--
#11	540.4	75	3.46	67.57	28.97	--

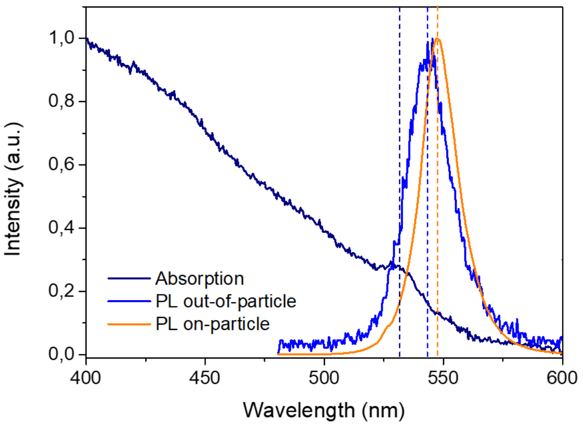

Figure S3. Normalized absorption and PL (out- and on- particle) spectra of sample A.

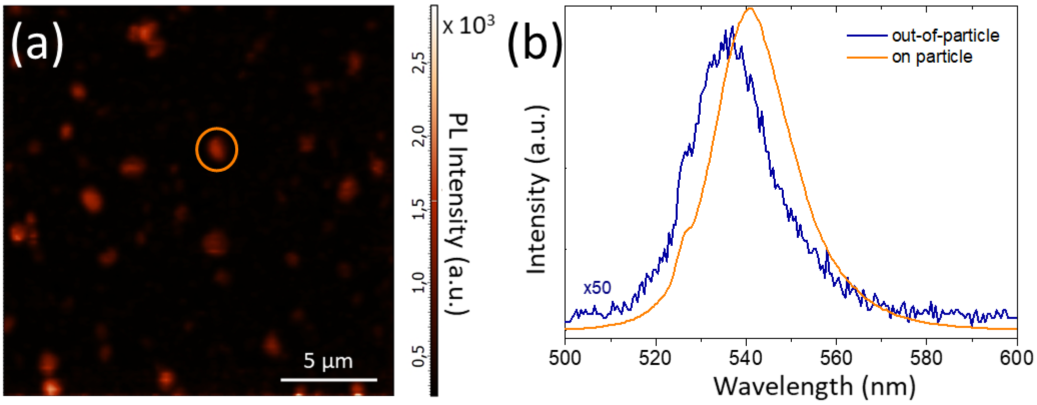

Figure S4. (a) Micro-PL intensity map of sample B acquired in reflection mode, using 470 nm laser light as an excitation source. (b) PL spectra acquired on particle (signed with an orange circle in panel a) and out-of-particle.

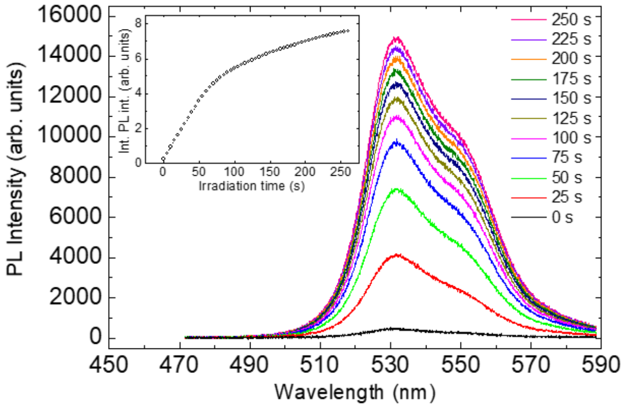

Figure S5. PL spectra of sample B during continuous UV irradiation in air. The main peak at about 532 nm, due to free EXs emission, and the shoulder at about 550 nm, due to localized EXs emission, are clearly visible. Inset: integrated PL intensity as a function of the irradiation time, evidencing the progressive intensity increase.
